# Myosin 5a in the Urinary Bladder: Localization, Splice Variant Expression, and Functional Role in Neurotransmission

**DOI:** 10.3389/fphys.2022.890102

**Published:** 2022-07-01

**Authors:** Josephine A. Carew, Vivian Cristofaro, Suhas P. Dasari, Sean Carey, Raj K. Goyal, Maryrose P. Sullivan

**Affiliations:** ^1^ Urology Research, VA Boston Healthcare System, Boston, MA, United States; ^2^ Harvard Medical School, Boston, MA, United States; ^3^ Brigham and Women’s Hospital, Boston, MA, United States

**Keywords:** bladder smooth muscle, protein splice variants, neurotransmission, myosin motor, peripheral nerve, Myosin 5a

## Abstract

Dysregulation of neurotransmission is a feature of several prevalent lower urinary tract conditions, but the mechanisms regulating neurotransmitter release in the bladder are not completely understood. The unconventional motor protein, Myosin 5a, transports neurotransmitter-containing synaptic vesicles along actin fibers towards the varicosity membrane, tethering them at the active zone prior to reception of a nerve impulse. Our previous studies indicated that Myosin 5a is expressed and functionally relevant in the peripheral nerves of visceral organs such as the stomach and the *corpora cavernosa*. However, its potential role in bladder neurotransmission has not previously been investigated. The expression of *Myosin 5a* was examined by quantitative PCR and restriction analyses in bladders from DBA (dilute-brown-nonagouti) mice which express a Myosin 5a splicing defect and in control mice expressing the wild-type *Myosin 5a* allele. Functional differences in contractile responses to intramural nerve stimulation were examined by *ex vivo* isometric tension analysis. Data demonstrated Myosin 5a localized in cholinergic nerve fibers in the bladder and identified several Myosin 5a splice variants in the detrusor. Full-length Myosin 5a transcripts were less abundant and the expression of splice variants was altered in DBA bladders compared to control bladders. Moreover, attenuation of neurally-mediated contractile responses in DBA bladders compared to control bladders indicates that Myosin 5a facilitates excitatory neurotransmission in the bladder. Therefore, the array of Myosin 5a splice variants expressed, and the abundance of each, may be critical parameters for efficient synaptic vesicle transport and neurotransmission in the urinary bladder.

## Introduction

The process of micturition provides periodic emptying of the bladder by simultaneous relaxation of the urethral sphincter and strong contraction of the detrusor. The latter is largely a consequence of local release of excitatory neurotransmitters from nerves permeating the detrusor. Within the bladder wall, intramuscular nerve bundles branch repeatedly into single fibers consisting of varicosities and intervaricose segments (Gabella, 1995). From varicosities formed along the axons, excitatory neurotransmission in the non-primate detrusor is accomplished primarily by exocytosis of the contents of acetylcholine- and ATP-containing synaptic vesicles. These neurotransmitters respectively activate muscarinic and purinergic receptors on bladder smooth muscle cells, inducing contraction. The complex process whereby neurotransmitter-containing vesicles are transported to the active zone in the varicosity membrane of bladder nerves has received little attention to date, although dysregulation of neuromuscular transmission may underpin a number of prevalent disorders of bladder function, including neurogenic, diabetic and obstructive urinary bladder dysfunction. In axons of the central nervous system, where neurotransmission processes have been extensively studied, the directed motion of synaptic vesicles along subcortical actin fibers is facilitated by the motor protein, Myosin 5a (Myo5a). The present study investigated the role of this motor protein in the regulation of excitatory neurotransmission in the bladder.

Myo5a is a processive motor that enables the short-range intracellular transport of molecular cargo along actin filaments. Expressed by many cell types specialized for secretion, Myo5a is a dimer composed of two heavy chains, each consisting of distinct domains that coordinate to carry out its functional roles. The N-terminus contains actin-binding and ATP-hydrolyzing motor regions, followed by a neck (or IQ domain) segment with six binding sites for Myo5a light chains, which include the calcium-sensing protein, calmodulin. The central portion contains the alpha-helical dimerization interface, consisting of three coiled-coil regions. This segment of heavy chain partially overlaps with a region of alternative exons (termed consecutively A, B, C, D, E, and F) which may occur in different spliced arrangements (Seperack et al., 1995; Lambert et al., 1998; Au and Huang, 2002; Wagner et al., 2006). Immediately following the alternate exon region is the C-terminal globular tail domain (GTD), which facilitates direct interactions with cargos to be transported.

The effects of *Myosin 5a* (*Myo5a*) genetic defects are often identified by pigmentation abnormalities, because Myo5a is required for melanosome transport in melanocytes. However, the more devastating consequences of Myo5a deletion or mutation are neurological. Homozygosity for the *Myo5a* null allele is a fatal trait associated with opisthotonus and convulsions leading to death in early infancy, as has been shown in the equine lavender foal, the dilute-opisthotonus and BD-IV rats, and the dilute-lethal mouse (Mercer et al., 1991; Futaki et al., 2000; Brooks et al., 2010; Landrock et al., 2018). In humans, *MYO5a* mutations cause the autosomal recessive disorder, Griscelli syndrome type I, in which hypopigmentation is coupled with cognitive impairment, delayed motor development, and hypotonia (Griscelli et al., 1978; Pastural et al., 1997). Interestingly, a variant of this syndrome was described with abnormal pigmentation as the only sign; the causal mutation was deletion of the segment encoding alternative exon F (Menasche et al., 2003; Yilmaz et al., 2014). Two *Myo5a* mutations that also cause exon F skipping have been characterized among non-lethal strains of dilute mice (Huang et al., 1998). Myo5a lacking exon F cannot bind well to and efficiently transport melanosomes, accounting for abnormal coloration in the absence of severe neurological defects in the affected Griscelli syndrome patients and the two dilute mouse strains. As the latter examples illustrate, not all *Myo5*a defects carry grievous neurological consequences. The homozygous dilute/brown/non-agouti (or DBA) mouse also has a non-lethal defect in *Myo5a* expression, which includes reduced pigmentation but not severe neurologic abnormalities.

The defect in DBA animals results from integration of the ectopic murine leukemia virus, Emv-3, within the intronic region of *Myo5a* between exons C and D, which disrupts normal mRNA splicing. While a fraction of transcripts produced in DBA mice carry exon C aberrantly spliced to the murine leukemia virus sequence and then prematurely truncated, such read-through transcripts are found more frequently in skin where exon C is often spliced to exon D, than in brain where exon C is predominantly spliced to exon E (Seperack et al., 1995). In the brains of DBA mice, the correct neuronal splice variant (ABCE) is expressed almost exclusively, and to an extent that is clearly sufficient to support normal neurological development and brain function.

In contrast to its expression in skin and brain, *Myo5a* expression in the peripheral nervous system has received less study. However, Myo5a has been identified in enteric nerves (Drengk et al., 2000) as well as the peritracheal and peribronchial plexuses (Buttow et al., 2006). Our previous work demonstrated that Myo5a is a constituent of peripheral nerves in the gastric *fundus* and *corpus cavernosum* (Chaudhury et al., 2011, 2012; Chaudhury et al., 2014). With respect to the bladder, Myo5a has been seen in the umbrella cells of the uroepithelium (Khandelwal et al., 2013) as well as in bladder tissue at both mRNA and protein levels, where its expression has been shown to increase then decline in the detrusor during the course of streptozotocin-induced diabetes (Yang et al., 2019). However, no study has examined the particular Myo5a splice variants expressed in bladder or the functional contribution of this motor protein to bladder neurotransmission. We recently demonstrated that multiple Myo5a splice variants exist in nerves of the myenteric plexus, embedded between the gastric longitudinal and circular muscle layers, as well as in their axons which course throughout the smooth muscle (Carew et al., 2021). The repertoire of Myo5a splice variants and their relative amounts in bladder nerves are likely to be adapted to the particular demands for molecular cargo transport that are relevant to neurotransmission in this organ. Therefore, in the present work we first analyzed expression of Myo5a splice variants, and then exploited the Myo5a deficiency of the DBA mouse to correlate the molecular characteristics of bladder Myo5a with the extent of excitatory neurotransmitter release from bladder varicosities, as reflected by detrusor contractile patterns.

## Materials and Methods

### Animals

All animal procedures were approved by the Institutional Animal Care and Use Committee of the VA Boston Healthcare System. Age-matched male C57/BL6 (RRID:IMSR_JAX:000664) and DBA/2J (RRID:IMSR_JAX:000671) mice were obtained from Jackson Labs (Bar Harbor, ME, United States). Following euthanasia by CO_2_ asphyxiation, urinary bladders intended for *ex vivo* functional studies were quickly removed and placed in cold Kreb’s solution (NaCl 120mM; KCl 5.9mM; NaHCO_3_ 25mM; Na_2_H_3_PO_4_ 1.2mM; MgCl_2_ • 6H_2_O 1.2mM; CaCl_2_ 2.5mM; dextrose 11.5 mM). The base of the bladder was removed and the mucosa was separated from the detrusor by sharp and blunt dissection under a stereomicroscope. Samples of the detrusor (*muscularis propria*) intended for mRNA analyses were stabilized by immediate immersion in RNA-later solution (Sigma-Aldrich, St Louis, MO, United States), while those intended for protein analyses were snap-frozen and stored at −80°C until extraction. In addition, the cortical region of the brain, a portion of skin, and the bladder mucosa were obtained from each animal, stabilized in RNA-later or snap-frozen, then analyzed in some aspects of this study. Bladder tissue with intact mucosa to be used for histology was fixed in Bouin’s solution (7 h, room temperature) and embedded in paraffin. Tissue intended for confocal microscopy was embedded in OCT compound and immediately snap frozen.

### Histology

Full thickness bladder tissue sections from WT (*N* = 3) and DBA (*N* = 3) mice were cut on a microtome and processed with hematoxylin and eosin (H&E) or Masson’s Trichrome (MT) stains according to standard procedures, which were performed at the Rodent Histopathology (DF/HCC) Core, Harvard Medical School. In tissue sections stained with MT, images were acquired from at least 6 regions of separate sections from each bladder from each strain and the areas of collagen and smooth muscle were determined by ImageJ software. The ratio of collagen to smooth muscle was compared between WT and DBA bladders.

### Antibodies

Antibody to Myo5a was obtained from Sigma-Aldrich (St Louis, MO, United States; M4812-LF18, RRID:AB_260545, referred to as LF18). LF18 does not recognize a Myo5a target in the S91 cell line, which was derived from melanocytes of a Myo5a-null mouse (Seperack et al., 1995), although it does recognize a target protein present in normal melanocytes and in HeLa cells, which is diminished in the presence of Myo5a-targeting siRNAs (Lindsay and McCaffrey, 2011). Antibodies to the internal control protein, β-actin (sc-47778, RRID:AB_2714189), to the muscarinic M_3_ and M_1_ receptors (M_3_R, sc-9108, RRID:AB_2291779; M_1_R, sc-365966, RRID:AB_10847359), to vesicular acetylcholine transporter (VAChT, sc-7716; RRID:AB_2188145), to the urothelial marker Uroplakin-3 (UPIIIa, sc-166808, RRID:AB_2241422), and to the smooth muscle marker myosin heavy chain (Myh11, sc-98705, RRID:AB_2282267) were obtained from Santa Cruz Biotechnology, Santa Cruz CA, United States). Antibodies to the neural markers synaptophysin (ab72242, RRID:AB_1271107) and peripherin were from Abcam (Cambridge, MA, United States) and Chemicon International (AB1530, RRID: AB_90725). The P2X_1_ purinergic receptor (P2X_1_R) antibody (APR-001, RRID:AB_2040052) was from Alomone Labs (Jerusalem, Israel). Horseradish peroxidase (HRP)-conjugated secondary antibodies for Western blotting were obtained from Santa Cruz (sc-2357, RRID:AB_2687626; sc-2357,RRID:AB_628497; sc-2354, RRID:AB_628490), while AlexaFluor-conjugated secondary antibodies for confocal microscopy were from Thermo-Fisher Scientific (Waltham, MA, United States: A11011, RRID:AB_143157; A21202, RRID:AB_141607; A21467, RRID:AB_10055703).

### Laser Scanning Confocal Microscopy

Full thickness WT bladder sections were cut on a cryostat (10–12 µm) and fixed with cold acetone for 10 min. After rinsing in phosphate buffered saline (PBS), sections were blocked with 5% donkey serum and 0.05% triton X-100 in PBS for an hour, then incubated overnight with rabbit primary antibody against Myo5a (1:500 dilution.) After extensive washing in PBS, sections were incubated with fluorescent secondary antibody diluted in PBS for 2 h at room temperature. Sections were double labeled with either mouse anti-synaptophysin (1:10) or goat anti-VAChT antibody (1:200) followed by anti-mouse or anti-goat AlexaFluor 488 (1:2000), respectively. Secondary antibodies were affinity-purified to reduce cross reactivity with endogenous or off-target species of immunoglobulins. On other tissue sections, Alexa Fluor 488 conjugated-UPIIIa (1:250) was used to mark the urothelial layer. Control slides in which the primary antibodies were omitted from preparations were processed in parallel. Sections were mounted using Fluoromount-G (Southern Biotech, Birmingham, AL, United States) and examined by confocal microscopy (Zeiss, LSM 710). For image acquisition of double-labelled tissue, slides were scanned separately at each excitation wavelength to minimize emission crosstalk.

### RNA Extraction and Analyses

Total RNA purification was carried out using the Qiagen RNeasy Plus Mini kit according to the manufacturer’s directions. Tissues were disrupted in buffer RLT plus ß-mercaptoethanol using the Tissue Lyser II mill-bead apparatus (Qiagen, Gaithersburg, MD, United States) by two cycles of 2 minutes each at 30 Hz with a 5 mm stainless steel bead. RNA concentration and purity were determined by ultraviolet spectrophotometry.

To prepare total RNA for reverse-transcriptase realtime PCR (RT-PCR), 100 ng aliquots were converted to cDNA using the High-capacity RNA to cDNA kit and random octamer primers. The cDNA was then tested with universal PCR mastermix and TaqMan primer/probe sets Mm00487823_m1, Mm01146036_m1, Mm01170524_m1 and Mm01146042_m1 which detect, respectively, the N-terminal motor domain, exon B, exon F, and the C-terminal globular tail domain of murine Myo5a. All reagents were from Thermo-Fisher Scientific (Waltham, MA, United States). RT-PCR was performed on an Applied Biosystems QuantStudio 6 Flex under a standard protocol of 40 amplification cycles of 15 s at 94°C and 60 s at 60°C. Each cDNA sample was assayed in triplicate for the primer/probe sets, and normalized using an internal control reaction measuring 18S RNA. No-template controls were performed for each assay, and the results were analyzed by the ∆∆Ct method for comparative gene expression.

For standard PCR, cDNA was prepared from 100 ng total RNA using an oligo-dT primer and the Superscript IV (VILO) kit (Thermo-Fisher Scientific). cDNA aliquots were used as templates for PCR using a forward primer within Myo5a exon A and reverse primer within the GTD. Products from this initial PCR with primer pair 1 (see Table 1 for sequences) were purified with the Qia-quick PCR-purification kit (Qiagen), then used as templates for nested PCR with internal primers (pairs 2, 3 and 4. Table 1). Twenty-five cycles of PCR under standard conditions were performed in an Eppendorf thermal cycler (Hauppauge, NY, United States) using the Taq PCR mastermix (Qiagen). PCR products were assessed in comparison to mass standards before and/or after digestion with restriction enzymes (Sty I or Ban II, New England Biolabs, Ipswich, MA, United States), by electrophoresis in Tris/acetate/EDTA buffer, on 2% MetaPhor agarose gels (Lonza, Rockland, ME United States) containing fluorescent Sybr-green dye. Gels were imaged using an Amersham Imager 600 (General Electric, Pittsburgh, PA, United States). The fluorescence intensity of each band was measured and corrected for fragment size, then the percent of fluorescent signal present in each band was calculated for each lane, and the averages for WT and DBA detrusors were determined.

**TABLE 1:** Primers used for Myo5a PCR.

Primer pair	Forward (5′-3′)	Reverse (5′-3′)
**1**	GCA​GAA​ACT​GAA​GAC​ATT​GCA​C	TCA​GAT​TGA​CAG​CCA​CAC​CA
**2**	ACT​AGA​GTA​TGA​GTC​TCT​CAA​GCG	TCA​GAT​TGA​CAG​CCA​CAC​CA
**3**	ACT​AGA​GTA​TGA​GTC​TCT​CAA​GCG	TCA​GCC​GGG​TGA​TCT​CAT​GCT​GCA​GG
**4**	ACT​AGA​GTA​TGA​GTC​TCT​CAA​GCG	TGA​TAC​TTC​TTA​GGG​TCA​TCG​C

### Protein Extraction and Western Blotting

Tissues were homogenized in RIPA buffer (Santa Cruz Biotechnology) supplemented with protease- and phosphatase-inhibitor cocktails (Sigma Aldrich), using the Tissue Lyser II apparatus by two cycles of 3 minutes each at 30 Hz with a stainless-steel bead. Following centrifugation to remove insoluble materials, total protein concentration was determined with the bicinchoninic acid protein assay (Sigma Aldrich) by measuring the absorbance at 280 nm with a biophotometer (Eppendorf). Samples of equivalent protein concentration were directly compared by electrophoresis, which was performed on Nu-PAGE (either 3–8% Tris-acetate or 4–12% Bis-Tris gels) followed by transfer to nitrocellulose membranes using the iBlot II semi-dry apparatus (Thermo-Fisher Scientific). To prevent non-specific antibody binding, blots were blocked in Tris-buffered saline plus 0.05% Tween-20 with 5% dry milk. Membranes were incubated overnight at 4°C with primary antibodies, then after extensive washing were incubated for 1 h at room temperature with HRP-conjugated secondary antibodies. After further washing, protein bands were developed on the membranes with Western-Lightening ECL reagents (Perkin-Elmer, Waltham, MA), and visualized using the Amersham Imager 600.

### Functional Analyses

After the bladder base and mucosa were removed, bladder smooth muscle tissue from each WT and DBA mouse was cut into longitudinal strips and mounted for isometric tension analysis, as described previously (Cristofaro et al., 2012). Briefly, each strip was connected to a force transducer (Grass Instruments, Middleton, WI, United States) on one end and to a fixed hook on the other end. Tissue was mounted between platinum electrodes in organ baths containing Krebs’s solution maintained at 37°C and bubbled with a mixture of 95% O_2_ and 5% CO_2_. Bladder tissue was stretched to passive tension of 0.5 g and equilibrated for at least an hour. Data was acquired at 40 Hz by DataQ data acquisition system driven by WinDaq software.

Neurally-mediated contractions were induced by electrical field stimulation (EFS) over a range of frequencies (1–64 Hz, 40 V, 0.5 ms pulse width, 10 s duration). Features of the resulting contractions were defined by the following parameters: maximum amplitude, area under the curve (AUC), slope (the first derivative of the rising phase, dF/dT), time to peak (interval between start of contraction and peak force), as well as tau (time constant of a decaying exponential fit to the descending phase of the contraction below 50% of the peak). EFS-induced contractions were repeated after cholinergic or purinergic inhibition. The cholinergic component of the contraction was inhibited with the muscarinic receptor antagonist atropine (1 µM) by pre-incubating the tissue for 30 min. To inhibit the purinergic component of the contraction, tissue was pre-incubated for 30 min with the selective purinergic receptor antagonists, NF449 (50 µM) and 5-BDBD (50 µM). The contractile responses to KCl (120 mM), α-β-methyl-ATP (10 µM) and carbachol (CCh, 10 µM), were also measured in each bladder strip. In some experiments, contractile responses to CCh were repeated in the presence of muscarinic M_1_ receptor antagonist, pirenzepine (0.1 µM). At the end of each experiment, the weight and length of each tissue strip was measured after blotting excess liquid on filter paper. Active stress was calculated as the measured force/cross-sectional area. NF449, 5-BDBD, CCh, α-β-methyl-ATP, atropine and pirenzepine were purchased from Sigma and Tocris (Minneapolis, MN, United States).

### Data Analysis

Differences between WT and DBA in band intensity on western blots, fold change in gene expression, or contractile responses were determined by Student’s *t*-test after testing for normality and equal variance. If these assumptions were not met, a Mann-Whiney *U* test or a Welch’s *t*-test, respectively was used. Differences in EFS responses between groups before and after antagonists were analyzed by a repeated measures two-way ANOVA followed by Holm-Sidak test for multiple comparison. Data are presented as mean ± SEM, and *p* < 0.05 were considered significant.

## Results

The animal strains used for these studies, C57/BL6 (homozygous, expressing the wild-type *Myo5a* allele, and referred to as WT) and DBA/2J (homozygous, expressing a mutated *Myo5a* allele, and referred to as DBA) were closely matched in general characteristics. The mean age of WT animals was 20.2 ± 1.7 weeks old, which was not significantly different from that of DBA animals (21.8 ± 1.7 weeks old). In addition, the average body weight (30.38 ± 1.03 g) and bladder weight (0.0280 ± 0.009 g) of WT animals were not different from the average body weight (30.03 ± 0.88 g) and bladder weight (0.0296 ± 0.002 g) of DBA animals.

### Myo5a is Expressed in the Bladder and Localized on Intramural Nerve Fibers

**FIGURE 1:**
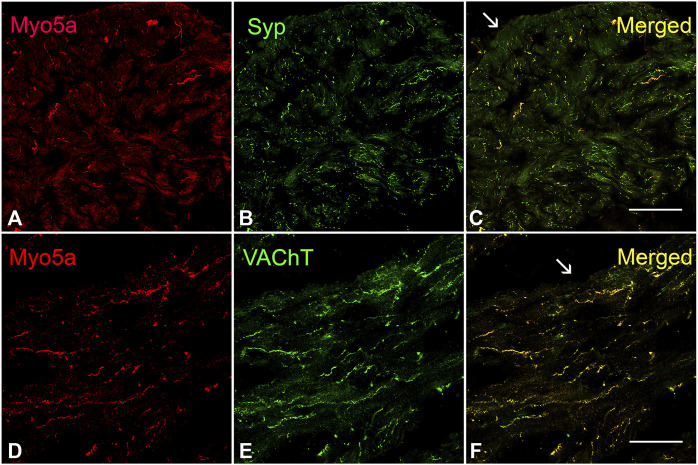
Expression of Myo5a in bladder tissue. **(A–C)** Laser scanning confocal microscopy of sections from WT detrusor detected Myo5a immunoreactivity (red staining) on nerve fibers that were immunoreactive for the pan-neuronal marker synaptophysin (Syp, green staining). The merged image on the right shows co-localization of these markers in yellow. **(D–F)** Immunoreactivity for Myo5a (red staining) and vesicular acetylcholine transporter (VAChT, green staining) in WT bladder *muscularis*. Co-localization of these two proteins in the merged panel (yellow) indicates expression of Myo5a in cholinergic fibers of the urinary bladder. Arrows indicate serosa. (Scale bar = 50 µm; Mag ×40).

The presence of Myo5a protein in the WT bladder, examined by confocal microscopy, was indicated by abundant immunoreactivity for Myo5a distributed throughout the bladder wall. As demonstrated by the co-localization of Myo5a staining with immunoreactivity for the varicosity marker, synaptophysin (Syp), as well as with the specific cholinergic varicosity marker, vesicular acetylcholine transporter (VAChT), Myo5a expressed in the detrusor layer was predominantly localized in cholinergic nerve fibers (Figure 1). No immunofluorescence was detected in negative controls (Supplementary Figure S1). Positive immunoreactivity for Myo5a was also detected within the urothelial and sub-urothelial layers (Supplementary Figure S2).

**FIGURE 2:**
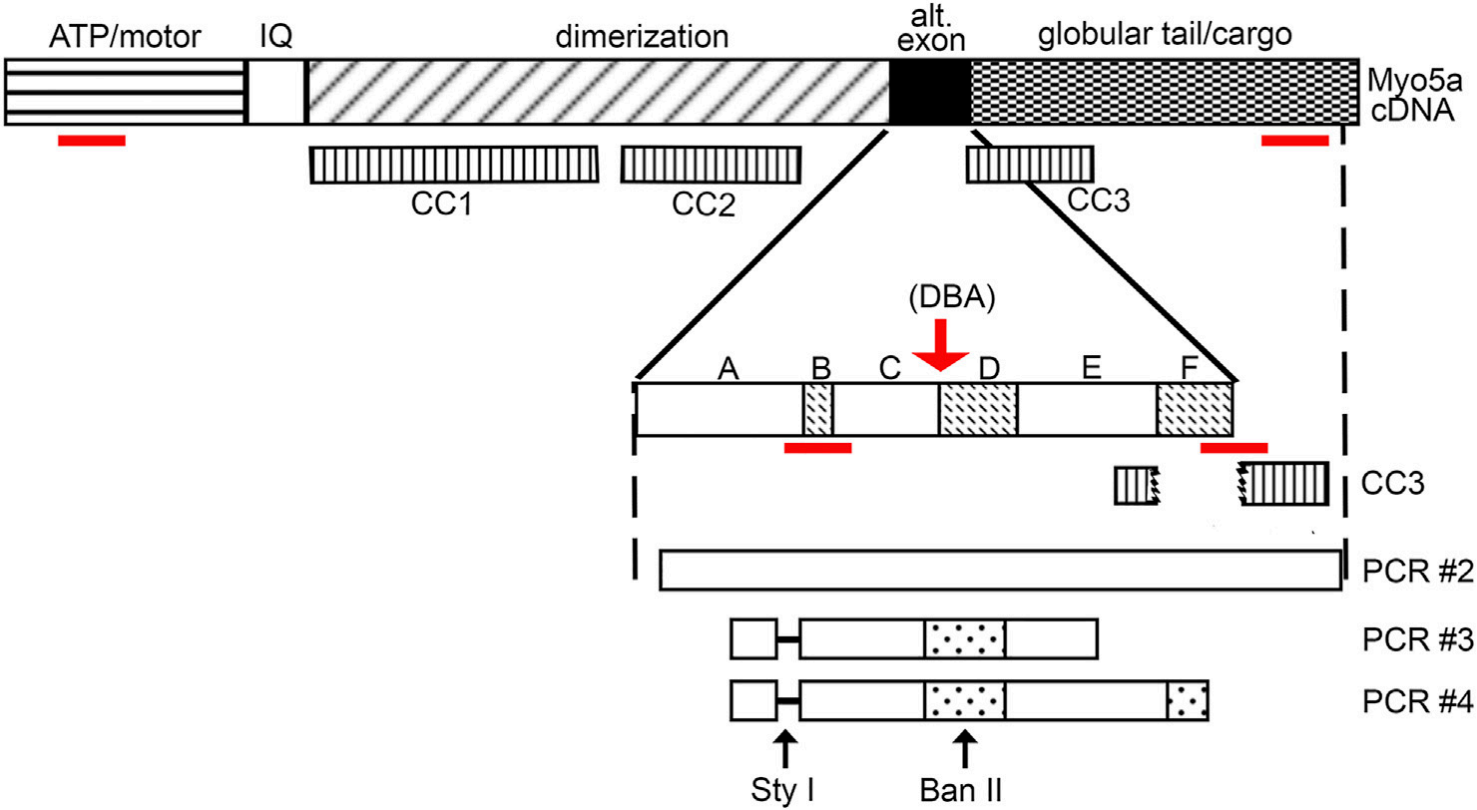
Myo5a schematic. Schematic of Myo5a mRNA/cDNA, depicting regions encoding the major structural domains of the Myo5a protein monomer: the ATP hydrolyzing/actin binding motor domain, the light-chain binding IQ domain, the central dimerization domain, the alternative exon domain, and the globular tail/cargo binding domain. Three coiled-coil regions (CC1, CC2, and CC3) have been described and are shown in the second line; CC1 and CC2 are within the dimerization domain, while CC3 is intact only in variants that do not contain exon F (such as the predominant brain form of Myo5a, ABCE), since it includes part of exon E sequence and part of globular tail domain sequence. CC3 is bisected by exon F, if present. Alternative exons are shown in the expansion; sequence encoding the constitutive exons A, C and E and the optional exons B, D and F are indicated by solid and speckled patterns, respectively. The red arrow indicates the position of the molecular defect in the *Myo5a* gene in the DBA mouse, which impairs mRNA splicing. Below, the products of detrusor nested PCR reactions are shown, as are the locations of restriction enzyme cleavage sites. The absence of exon B (and its diagnostic Sty I restriction site) in detrusor nested PCR products is indicated by the dashed line. The red lines indicate the positions of the RT-PCR assays employed.

**FIGURE 3:**
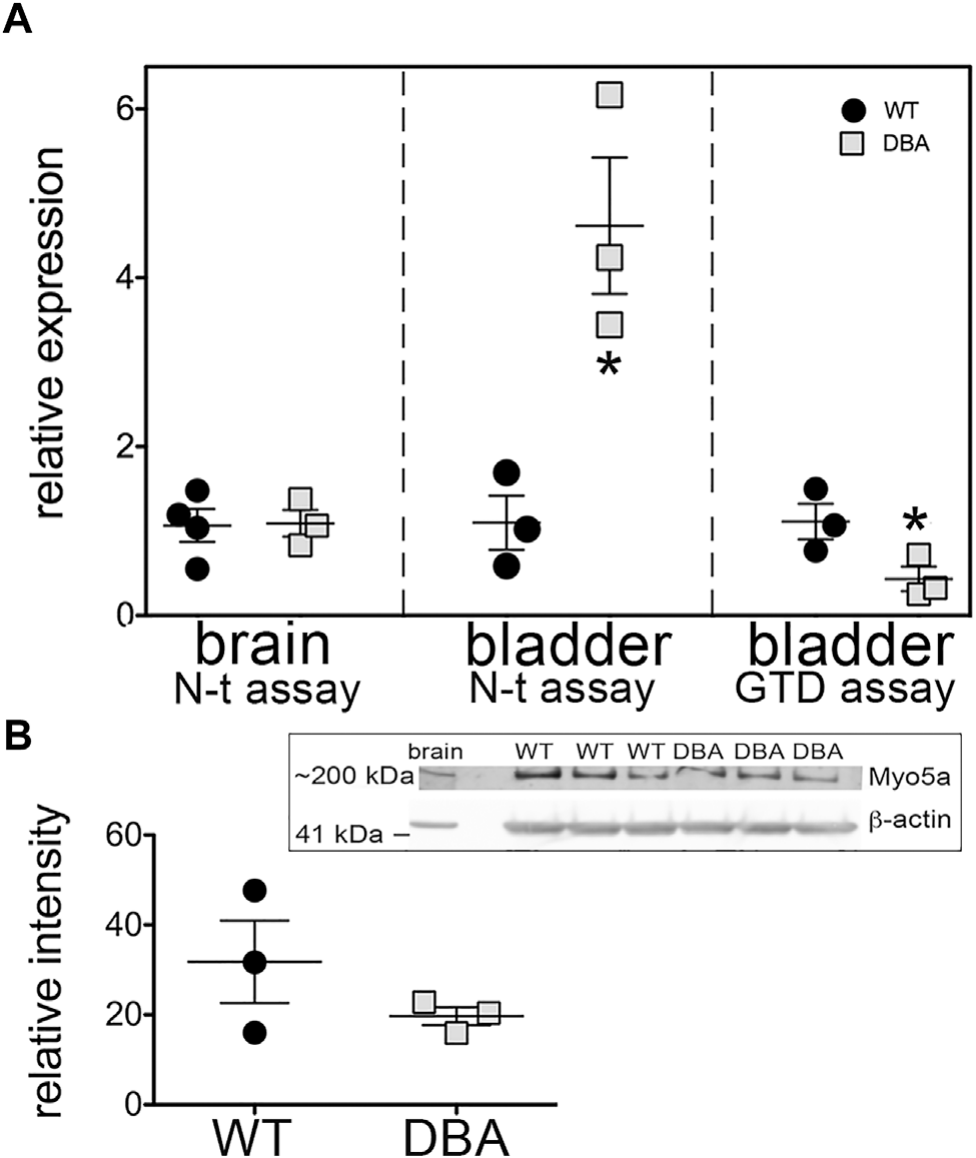
Overall Myo5a expression in bladder. **(A)** cDNA from brains and detrusors of WT (black circles, *N* = 3) and DBA animals (gray squares, *N* = 3) was assayed in triplicate for the two different pan-Myo5a RT-PCR primer/probe sets indicated, and for 18S rRNA as internal control. Expression was determined by the ∆∆Ct method and graphed relative to WT within each organ. Comparison of WT vs. DBA brain was not significant (*p* = 0.67), but of WT vs. DBA detrusor was significantly higher for the N-terminal (N-t) assay and lower for the globular tail domain (GTD) assay (*p < 0.05). **(B)** Representative immunoblot (inset box) and quantitative graph showing the expression of Myo5a protein in lysates from WT (black circles, *N* = 3) and DBA detrusors (gray circles, *N* = 3). 30 µg aliquots of detrusor extracts were immunoblotted with Myo5a antibody (LF-18). Brain extract was loaded in lane 1 as a positive control. When intensities of the ∼200 kDa Myo5a band were corrected for intensities of the 42 kDa ß-actin loading control, WT and DBA Myo5a protein levels were not significantly different (*p* = 0.4).

To address whether the *Myo5a* gene was expressed to the same extent in DBA as in WT bladders, the relative levels of Myo5a mRNA and protein were determined in those tissues by RT-PCR and immunoblotting, respectively. The relative regions of the Myo5a transcript sequence identified by two Taq-Man assays are widely separated and common to all potential full-length splice variants, as shown in Figure 2. The pan-Myo5a N-terminal assay spans the border between exons 8-9 within the region encoding the motor domain, while the pan-Myo5a GTD assay spans the border between exons 33–34 within the GTD. As shown in Figure 3A, *Myo5a* expression measured with the N-terminal assay was significantly higher in DBA detrusor compared to WT detrusor. Inversely, the level of expression measured with the GTD assay was significantly lower in DBA bladder than in WT bladder. However, *Myo5a* expression in WT versus DBA brains measured with the N-terminal assay was not different. Taken together, these data suggested reduced full-length Myo5a transcripts, despite increased transcriptional initiation in DBA detrusor compared to WT detrusor.

Anticipating that expression of Myo5a protein would be similarly decreased in DBA compared to WT detrusors, immunoblotting was performed with the same antibody used for Myo5a detection in Figure 1. The epitope for antibody LF-18 is residues 1782–1799 within the GTD, which is present in all Myo5a splice variants. In tissue lysates from both WT and DBA detrusor, a protein band of electrophoretic mobility consistent with the predicted Myo5a molecular weight (∼200 kDa) was detected. Because alternative exons B, D, and F are small (3, 27, and 25 amino acids respectively, compared to 1850 amino acids for Myo5a without these exons), it was expected that different variant proteins would be difficult to resolve from one another by gel electrophoresis. However, and in contrast to the Myo5a mRNA expression findings, Myo5a protein band intensities were not different between WT and DBA detrusors (Figure 3B). The reason for this result is not known, but was somewhat surprising, since the RT-PCR results of Figure 3A had implied that, in DBA detrusor, a significant fraction of Myo5a transcripts likely contained exon C incorrectly spliced to the ectopic Emv-3 sequence rather than to exon D or exon E, producing incomplete transcripts which could not be translated into a functional protein.

### Expresssion of Myo5a Exons D and F in Bladder Varies by Genotype

Intriguingly, the discrepancy between WT and DBA detrusors, but not brains, with regard to total Myo5a measured by the N-terminal pan-Myo5a assay, also implied that non-brain Myo5a splice variants were expressed among the full-length Myo5a transcripts of bladder nerves. To examine detrusor Myo5a for the presence of alternative exons B, D and F, additional RT-PCR, as well as standard PCR experiments (schematized in Figure 2), were carried out.

The relative expression of Myo5a mRNA in WT versus DBA detrusor was compared using TaqMan assays targeted within the alternative exon region of the Myo5a transcript. One assay employed spans exon B while the second is directed against the exon F/GTD junction. Because these assays positively identify the hallmark exons of “brain” (exon B) and “skin” (exon F) among Myo5a transcripts, samples from skin and brain were concurrently assessed as controls for detrusor. (Data obtained in parallel for bladder mucosa are presented in Supplementary Figure S2)

**FIGURE 4:**
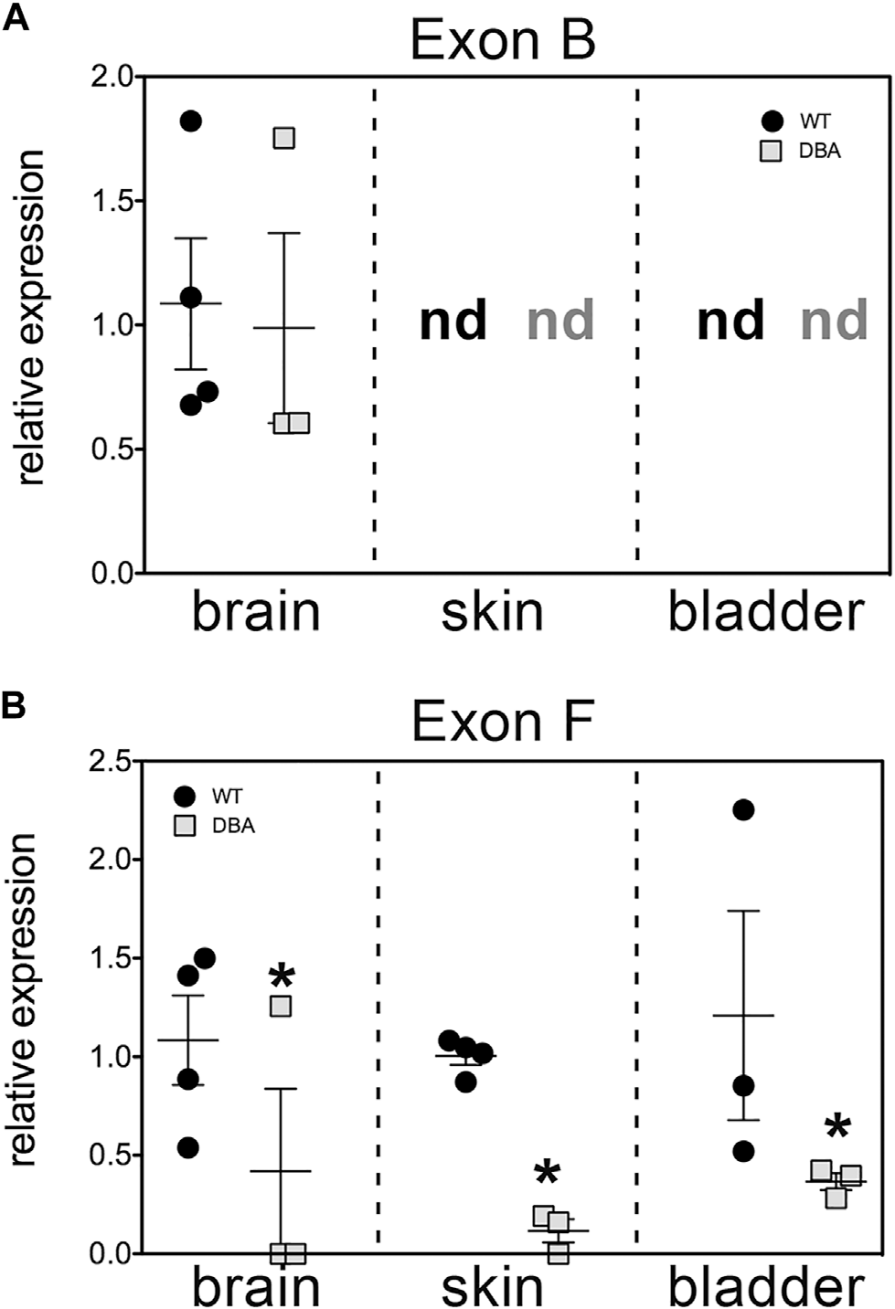
Relative expression of variant exons in WT and DBA tissues. cDNAs made from total RNA of brain, skin and detrusor of WT animals (black circles, *N* = 4) and DBA animals (gray squares, *N* = 3) were assayed in triplicate for Myo5a exon B or for exon F with their specific TaqMan assays, and for 18S rRNA as an internal control. Expression was determined by the ∆∆Ct method and graphed relative to WT within each tissue. Horizontal line indicates average relative expression ±SEM. **(A)** Expression of exon B in brain was not different in DBA compared to WT (*p* = 0.67). In detrusor and skin, exon B was not detected (nd) and therefore differences between strains could not be determined. **(B)** Expression of exon F was lower in DBA brain, skin and detrusor (*p < 0.05) than in corresponding WT tissues.

The results, shown in Figure 4, corroborate several key conclusions that have previously been established. Brain Myo5a mRNA overwhelmingly included exon B, and exon B expression was comparable in the brains of WT and DBA animals. Also, exon B was undetectable in skin, while exon F-containing variants were abundant in WT skin, but significantly reduced in DBA skin. However, the data also illustrated several unexpected features of Myo5a expression in the detrusor. First, exon B was not detected in bladder of either WT or DBA animals (Figure 4A). In contrast, exon F expression was seen in all WT tissues examined, including the brain (albeit at low levels). Furthermore, in comparison to the corresponding WT tissue, DBA detrusor evinced significantly reduced exon F expression (Figure 4B).

The hypothesis that DBA bladder was deficient in the larger Myo5a splice variants was also investigated by standard PCR and restriction enzyme analysis, a method which confers two advantages. First, it allows direct demonstration of the presence/absence of exon D (for which no RT-PCR reagents are available) and second, it permits amplification of fragments large enough to encompass multiple alternative exons, so that their linear arrangements can be discerned. Inspection of the sequence of murine Myo5a indicated that there are, within the common exons (A, C and E), a single consensus sequence apiece for restriction enzymes Sty I and Ban II (Figure 2). The nucleotide sequences encoding the alternative exons revealed that exon B introduces a second consensus Sty I site, while exon D introduces a second Ban II site. The susceptibility of PCR products to digestion and the relative sizes of the fragments generated with these enzymes (see Table 2) thus indicate compositions and linear arrangements of alternative exons in Myo5a bladder transcripts.

**TABLE 2:** Nested PCR products and digestion fragments.

Primer Pair	Exons	Uncut (bp)	Sty I (bp) exon B	Ban II (bp) exon F
**2**	+B + D + F	989	**246**, 649, 94	177, **228, 584**
	+B + D − F	914	**246**, 674, 94	177, **228, 509**
	+B − D + F	908	**246**, 568, 94	177, **656**
	+B − D − F	833	**246**, 493, 94	177, **731**
	−B + D + F	980	**886**, 94	177, **219, 584**
	−B + D − F	905	**811**,94	177, **219, 509**
	−B − D + F	899	**805**, 94	177, **722**
	−B − D − F	824	**730**, 94	177, **647**
**3**	−B + D	421	421	**200/221**
	−B − D	340	340	340
**4**	−B + D + F	475	475	**200/275**
	−B − D + F	394	394	394

PCR products generated using primer pairs 2—4 for the potential alternative exon sequence arrangements shown in the second column are given in base pairs (bp). Digestion of these products with restriction enzyme Sty I or Ban II, is predicted to generate fragments of the sizes shown. Digestion fragments which are common to digestion of all potential sequences are depicted in regular type; those which confirm the presence or absence of exon B or exon D in each context are shown in bold type. For PCR with primers pairs 3 and 4, sequences with exon B were not considered, since it is not detectable in detrusor.

**FIGURE 5:**
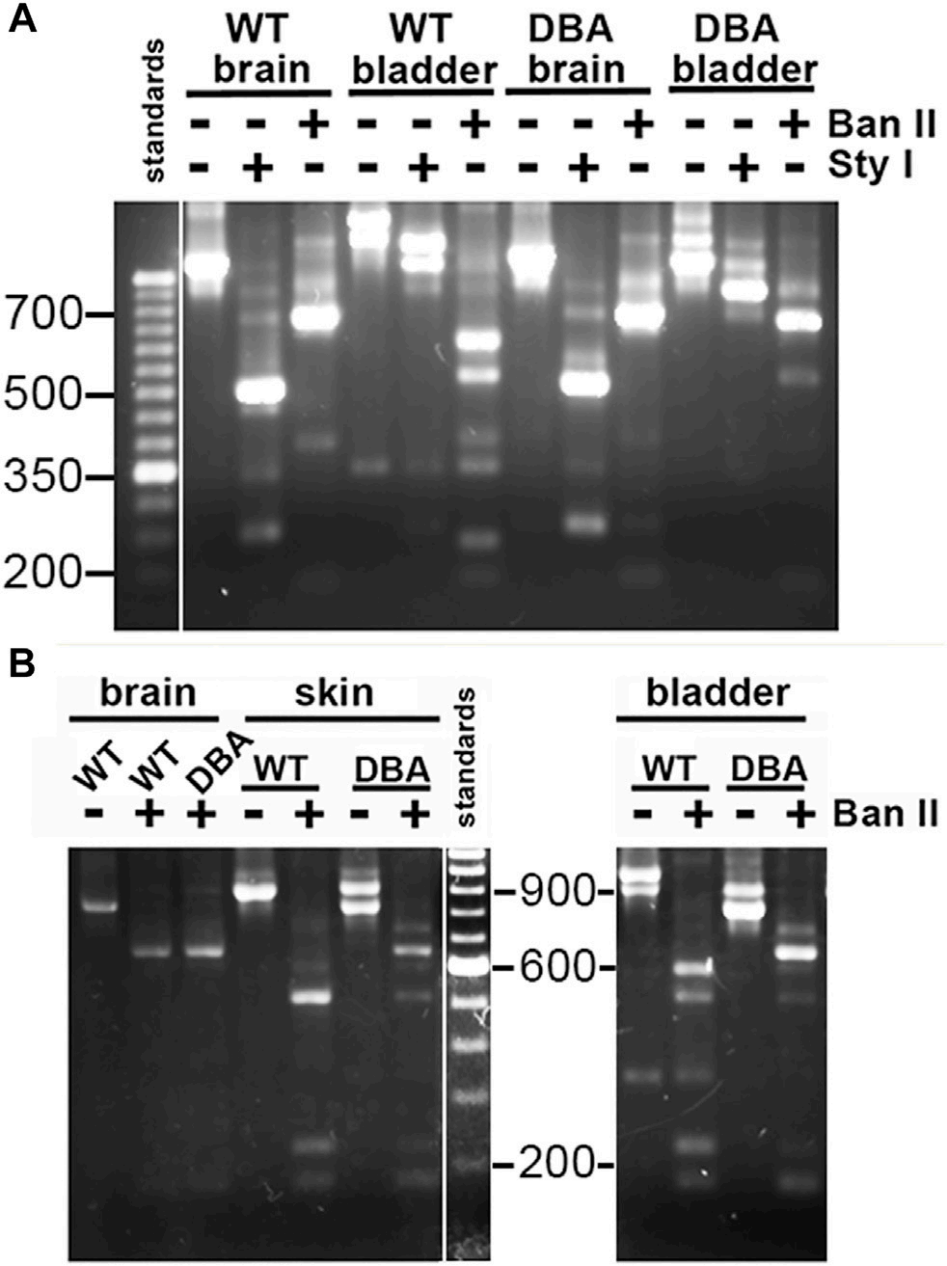
Myo5a splice variants in tissues of WT and DBA mice. **(A)** Illustration of differential Myo5a variant expression pattern in detrusor and brain from WT and DBA mice. Nested PCR fragments of brain and detrusor cDNAs from a WT and a DBA mouse pair were prepared with primer pair 2, then compared by agarose gel electrophoresis either undigested or digested with Sty I or Ban II, as shown above the lanes. A 50 base pair (bp) ladder with weighted 350 bp standard is at left. Contrast was adjusted separately for the standard lane, to bring out the positions of the smaller standards. **(B)** Nested PCR fragments prepared from brain, skin, and detrusor of a WT and DBA mouse with primer pair 2, were compared before and after digestion with Ban II. A100 bp ladder with weighted 600 bp standard is shown and contrast was adjusted separately. All lanes were from a single gel; those for detrusor PCRs and digests were moved to the right, so that sizes of the standard fragments could be added.

Primer pair 2 is predicted to generate nested PCR fragments spanning the entire alternate exon region, and ranging in size between 824 base pairs (bp) (for ACE, the smallest possible product) and 989 bp (for ABCDEF, the largest possible product). Figure 5A depicts the nested PCR products obtained from brain and detrusor cDNAs of a WT and of a DBA animal using this primer pair, as well as their restriction digestion patterns. The masses of the predominant WT and DBA brain products were identical. Since exon B is only 9 bp long, its presence cannot be inferred from uncut fragment mass alone. However, the brain product was cleaved by Sty I into fragment sizes indicative of exon B inclusion. When brain PCR products were digested with Ban II, no digestion product suggesting the presence of exon D or exon F was recovered, confirming their assignment as the expected arrangement, ABCE.

The same nested PCR performed on the WT and DBA detrusors presented a more complex pattern, with each genotype having at least two products giving rise to a mixture of digestion fragments. Confirming the RT-PCR results, Sty I digestion did not generate the 246 bp fragment diagnostic for exon B, which had been obvious in the digested brain PCR samples. In contrast, Ban II digestions were distinct according to strain. In the Ban II digest from WT detrusor, fragments suggesting arrangements ACDEF and ACDE among the initial products were evident. An approximately 250 bp fragment was identified, independently confirming inclusion of exon D in some WT detrusor products. However, the latter fragment was not visible in the Ban II digest of DBA detrusor products, where instead there was a prominent digestion fragment of approximately 750 base pairs, suggesting the presence of ACEF in the initial mixture (Figure 5A).

Exclusion of exon B from detrusor Myo5a, coupled with inclusion of exons D and F, suggested that the splice variants of bladder nerve Myo5a more closely resembled the pattern of skin than of brain. To explore this possibility, skin and brain from a second WT and DBA animal pair were used for nested PCR with primer pair 2, digested with Ban II, and compared to detrusor products by agarose gel electrophoresis (Figure 5B). Again, the brain digestion patterns indicated a single predominant product, consistent with arrangement ABCE. The WT detrusor digestion pattern indicated ample amounts of arrangements ACDE and ACDEF, while the DBA detrusor pattern indicated predominantly ACE with minor contributions from ACEF and ACDE. The WT skin pattern suggested that there was a predominant splice variant in this particular sample, and Ban II digestion identified it as ACDE, although fragments indicative of ACDEF and ACEF were also detected. Products from the DBA skin sample were digested by Ban II into fragments indicating that ACE was the most abundant variant amplified, with lesser amounts of ACEF, ACDE and ACDEF. Thus, Ban II digestion confirmed that Myo5a of WT skin included exons D and F, while DBA skin was deficient.

**FIGURE 6:**
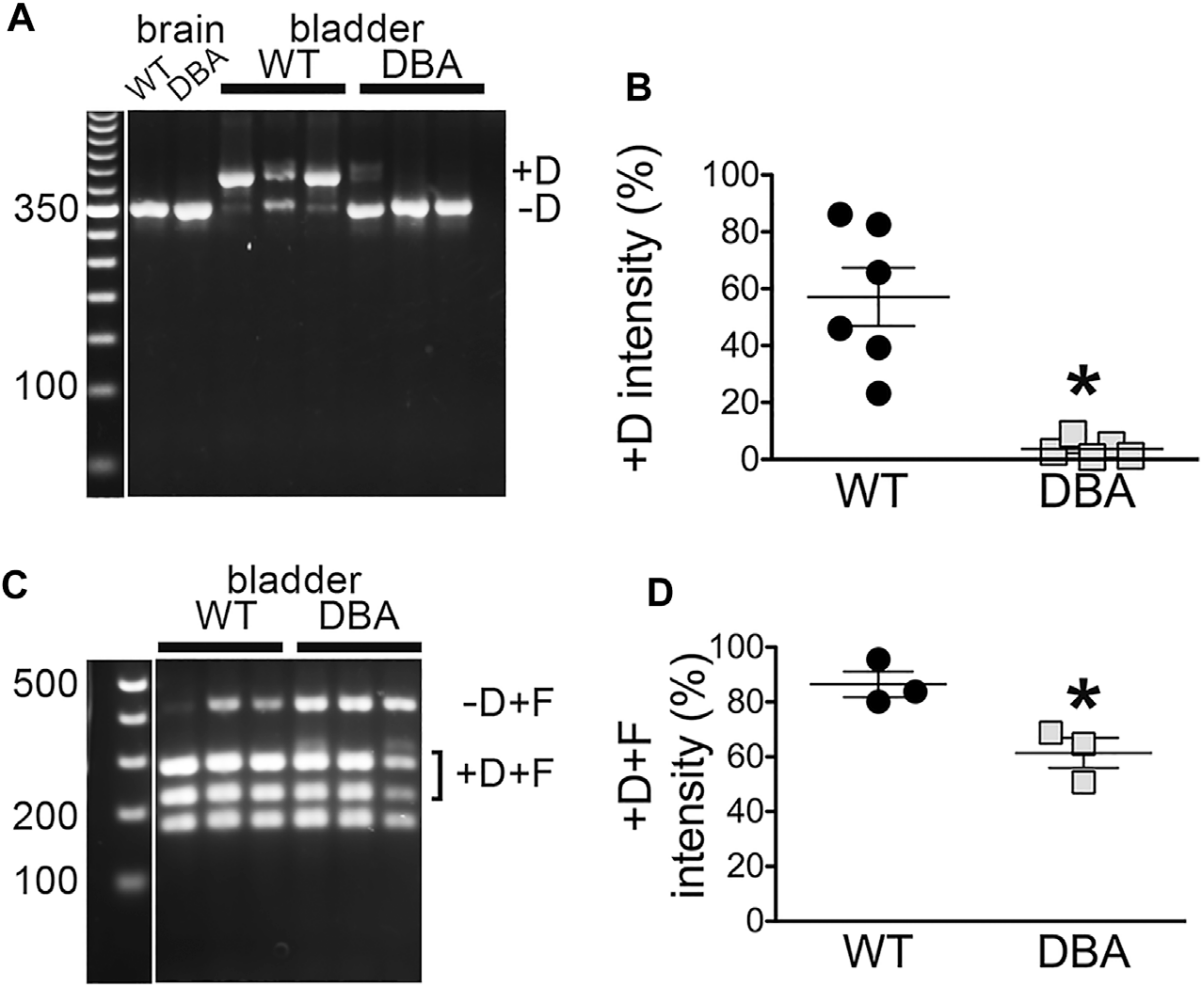
Myo5a exons D and F in bladders from WT and DBA mice. **(A)** Nested PCR fragments from detrusors of WT (*N* = 6) and DBA (*N* = 5) animals were prepared with primer pair 3 and electrophoresed. A representative agarose gel with bands corresponding to fragments with and without exon D from three WT and three DBA detrusors is shown, with individual WT and DBA brain samples as controls. Exposure of the 50 bp standard lane, with weighted 350 bp band, was adjusted separately. **(B)** The intensity of +D bands is graphed as a proportion of total intensity in each lane (sum of +D and −D) and plotted for WT (black circles), and DBA (gray squares). Horizontal line indicates average intensity in % ± SEM for the +D band in all replicates. The comparison between WT and DBA detrusor was significant (*p = 0.015). **(C)** Nested PCR fragments from detrusor of three WT and three DBA animals were prepared with primer pair 4, digested with Ban II to cleave the +D band, and electrophoresed. The relative positions of detrusor exon F-containing digestion fragments including or lacking exon D are indicated, and a flanking lane with 100 bp standards is marked. Contrast for the standard lane was adjusted separately. **(D)** Data are graphically represented; the horizontal line indicates average intensities in % ± SEM for PCR product containing exon D (black circles, WT; gray squares, DBA) are graphed. Coincidence of exons D and F in the same cDNA fragment was significantly reduced in DBA detrusor (*p* = 0.03).

To more rigorously interrogate multiple detrusor cDNA samples for inclusion of exon D, nested PCR was also done with primer pair 3 (Figure 6A), in which the forward primer is situated near the 3’ side of exon A and the reverse primer within exon E. Given the location of the primers, only two fragments were possible, differing in mass by the 81 bp encoding exon D. The amount of each fragment was quantitated as a fluorescent signal, corrected for fragment size, and compared to the other independent samples across the gel. Both the minus-exon D and plus-exon D products were observed in WT detrusor samples, but the plus-exon D product was significantly greater in WT compared to DBA detrusor (Figure 6B). Consequently, the minus-exon D product predominated in DBA detrusor samples.

The presence of exon D in PCR products that also included exon F was examined by nested PCR with primer pair 4, in which the forward primer sequence was again located within exon A but the reverse primer sequence was entirely within exon F (Figure 6C). In this reaction also, only two PCR products were possible, differing in undigested mass depending on the inclusion of exon D, which introduces a cleavage site for restriction enzyme Ban II. Cleavage of the larger, exon D-containing product generated a pair of smaller bands. The results indicated that in both WT and DBA detrusors, exon F was found predominantly in a linear arrangement with exon D. However, the proportion of the PCR product including both exons D and F was greater in WT detrusor (∼86%) than in DBA detrusor (∼62%), and this difference was significant (Figure 6D).

### Intrinsic Contractile Properties are Comparable in WT and DBA Detrusors

**FIGURE 7:**
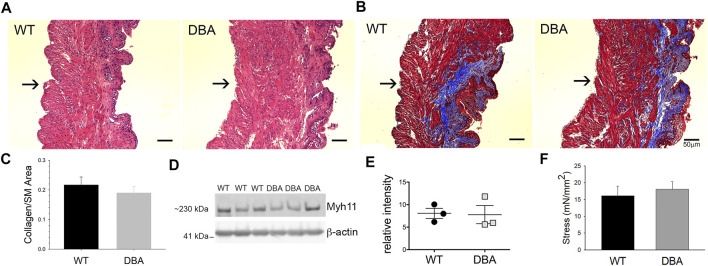
Morphologic, functional and molecular evaluation of bladder smooth muscle contractile apparatus. Histomorphometric evaluation of bladder tissue sections stained with H&E **(A)** and MT **(B)** from WT (*N* = 3) and DBA (*N* = 3) mice (Mag X10, scale bar 50 µm, arrows indicate serosa). **(C)** Quantification of the collagen/smooth muscle (SM) ratio was comparable between WT (black bar) and DBA (gray bar) bladders. **(D)** Representative Western blot and **(E)** quantitative graph of intensity of myosin heavy chain (Myh11) immunoreactivity detected in protein lysates from mucosa-denuded WT and DBA bladders. Band intensities in WT (black circles, *N* = 3) were not different from DBA (gray squares, *N* = 3) when normalized by ß-actin, which was used as the loading control. **(F)** Contractile responses induced by direct smooth muscle depolarization achieved by KCl 120 mM in WT (*N* = 11, black bar) and DBA (*N* = 13, gray bar) bladders were not different.

The histological features observed by H&E staining of bladder sections were comparable between WT and DBA mice. Tissue architecture, consisting of a distinct luminal epithelium, a collagen-rich lamina propria, and an outer layer of smooth muscle bundles appeared to be normal in DBA bladders (Figure 7A). Additionally, the ratio of collagen to smooth muscle area, determined by quantification of MT images, was not different between strains (Figures 7B,C). The expression of the smooth muscle marker, myosin heavy chain (Myh11), was not different between WT and DBA detrusors (Figures 7D,E). These findings confirm the absence of morphological abnormalities in DBA bladder such as fibrosis, hypertrophy, or contractile protein deficits that could negatively alter smooth muscle responses. The functional integrity of the intrinsic contractile machinery of DBA bladder smooth muscle was established by measuring isometric tension in response to a high potassium Kreb’s solution (HPK, 120 mM KCl). The amplitudes of contractions induced by HPK were not different between WT and DBA mice (Figure 7F).

### Contractile Responses to Nerve Stimulation are Impaired in DBA Mice

**FIGURE 8:**
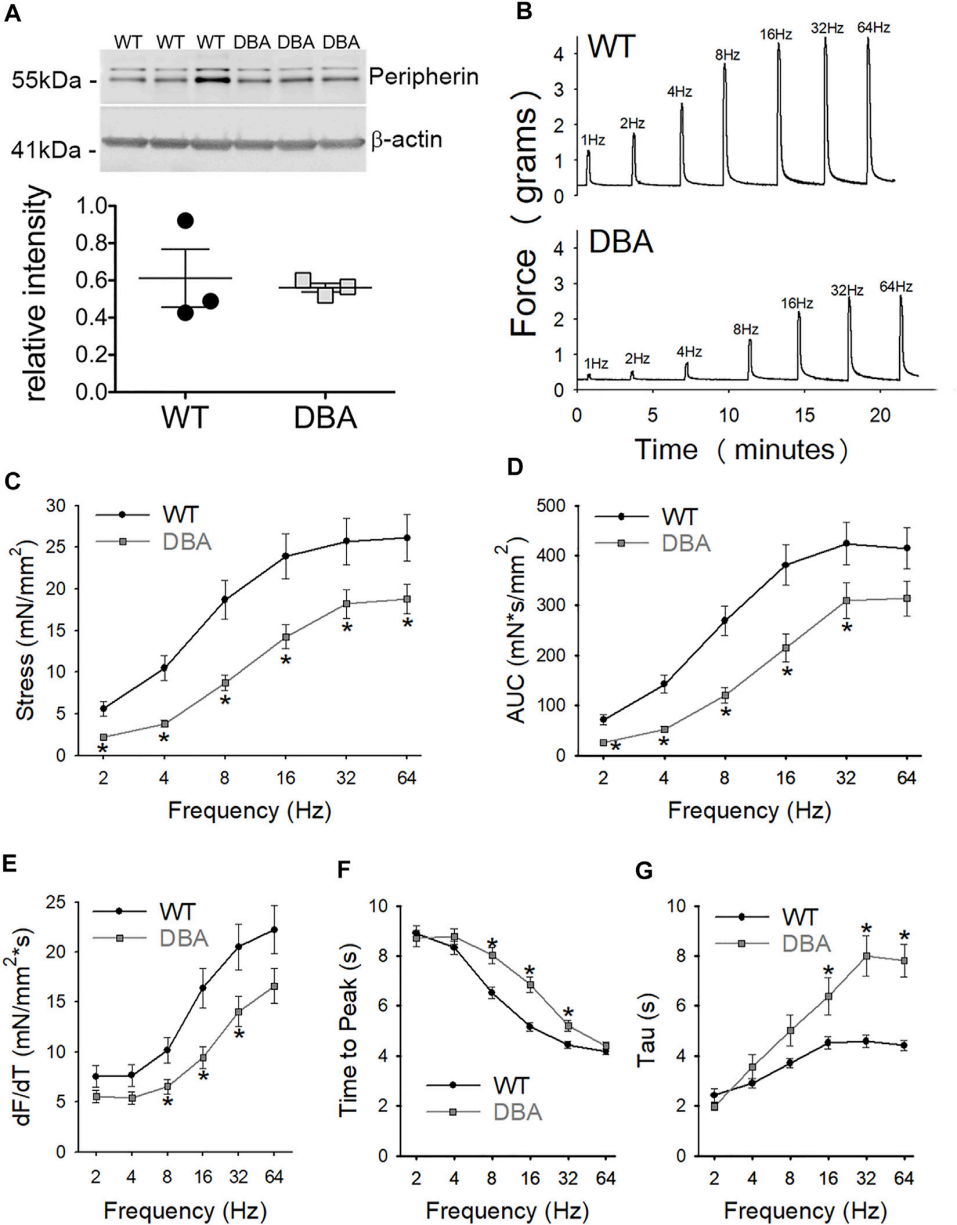
Contractile responses to EFS in WT and DBA bladders. **(A)** Representative membrane and quantitative graph showing the expression of neuronal marker peripherin in WT (*N* = 3, black circles) and DBA (*N* = 3, gray squares) detrusor lysates. The intensity of both immunoreactive bands, corresponding to the ∼56 and ∼58 kDa peripherin isoforms (Landon et al., 2000) were normalized by internal control β-actin. No differences in peripherin expression were detected between WT and DBA detrusors. **(B)** Representative tracing showing the contractile responses to EFS at frequencies between 2 and 64 Hz in WT (top trace) vs. DBA (bottom trace) detrusors. **(C)** The amplitude and **(D)** the area under the curve (AUC) of EFS-induced contractions of WT (black circles) and DBA (gray squares) detrusor tissue are plotted. Contractile responses of DBA detrusor were significantly lower than that of WT detrusor over a range of frequencies, as indicated by the asterisks. **(E)** The peak rate of rise of the ascending phase of the contraction, **(F)** the time to peak, as well as **(G)** the rate of decay (Tau) of the descending phase of the neurogenic contractions are graphed for WT (black circles) and DBA (gray squares) detrusors. Especially at middle to higher frequencies of stimulation, contractions in DBA mice were significantly decreased in slope with delayed time to peak and prolonged recovery time, compared to those measured in WT (*N* = 11 WT, *N* = 13 DBA; *p < 0.05).

Functional studies were performed to determine whether differences in Myo5a variant expression were associated with differences in neuromuscular transmission between WT and DBA detrusors. To determine whether the extent of detrusor innervation was comparable between strains, the expression of the nerve marker, peripherin (an intermediate neurofilament protein), was determined by Western blot. No difference in immunoreactivity for peripherin was detected in WT and DBA detrusor lysates (Figure 8A). Frequency-dependent force generation in response to EFS-induced nerve stimulation was detected in both WT and DBA detrusors (Figure 8B). However, the contractile responses to EFS in DBA detrusors were significantly lower in amplitude at all stimulation frequencies, and in AUC at frequencies of stimulation below 64Hz, compared to WT detrusors (Figures 8C,D). In addition, compared to WT, the frequency response curves in DBA detrusors were characterized by reduced rate of rise, longer time to peak (significant at 8–32 Hz), as well as slower rate of decay (significant at 16–64 Hz), (Figures 8E–G).

**FIGURE 9:**
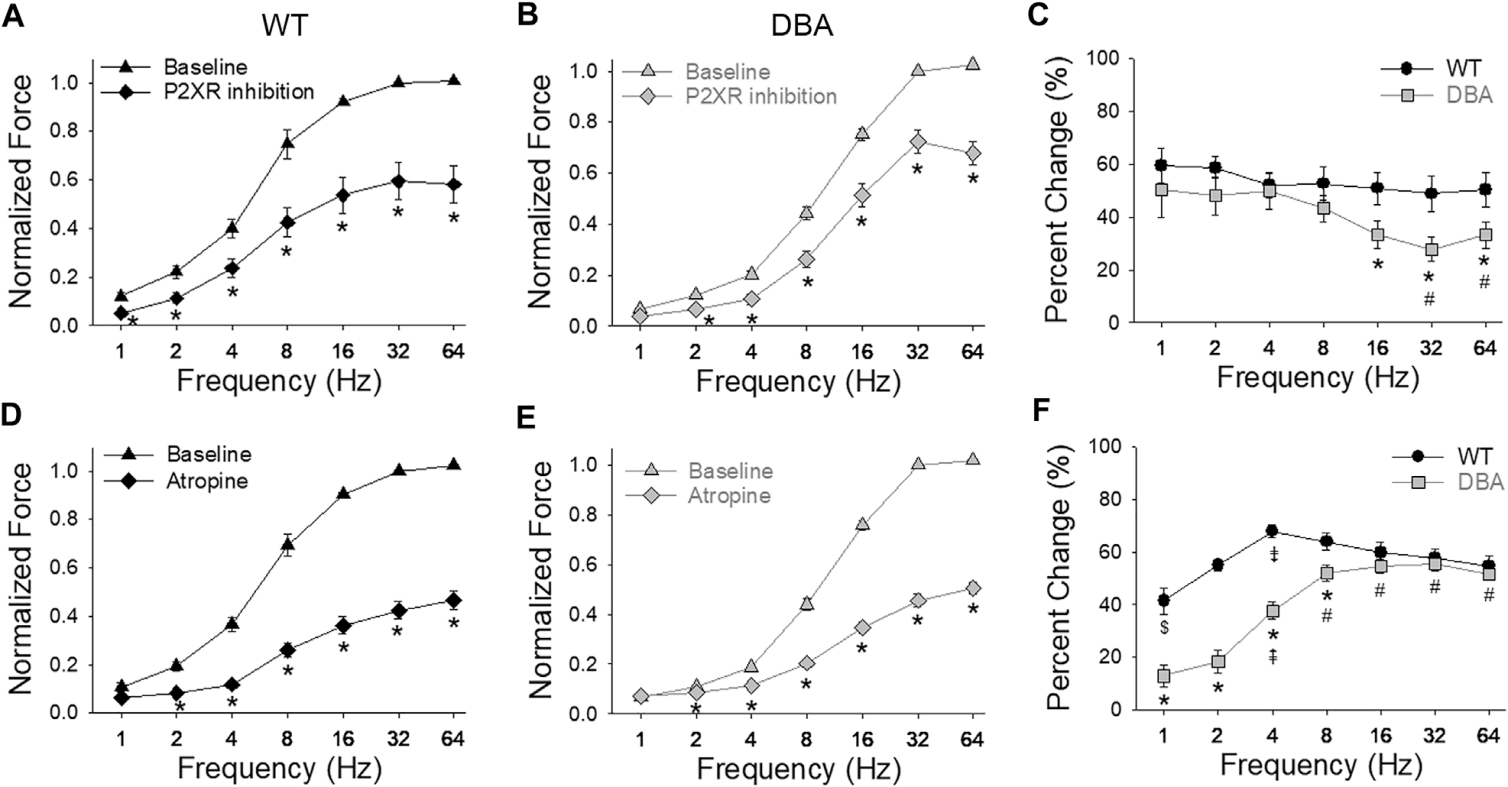
Purinergic and cholinergic inhibition of neurally evoked contractile responses. Effect of post-junctional inhibition on the frequency-response curves in WT [**(A,D)**, black symbols] and DBA [**(B,E)**, gray symbols] detrusors. Contractile responses to EFS in the absence (triangles) versus the presence (diamonds) of P2XR antagonists (NF449 and 5BDBD) in detrusors from **(A)** WT and **(B)** DBA mice. Following purinergic inhibition, responses to EFS of both WT and DBA detrusors were significantly reduced at all frequencies as indicated by asterisks. Data are normalized by the baseline response (without inhibitors) at 32 Hz. **(C)** Compared to WT (black circles), the reduction in force relative to the baseline response due to purinergic inhibition (change from baseline) was significantly lower in DBA detrusors (gray squares) at frequencies of stimulation above 8 Hz (*significantly lower than WT at this frequency; # significantly lower than 2 and 4 Hz within DBA). Comparison of the effect of muscarinic receptor antagonist atropine on nerve-mediated contractile responses in **(D)** WT and **(E)** DBA detrusors. Normalized force is graphed versus stimulation frequency, in the absence (triangles) and presence (diamonds) of atropine. Data are normalized by baseline responses at 32 Hz. Atropine significantly reduced the responses of both WT and DBA detrusor to EFS at frequencies greater than 1 Hz (*). **(F)** The inhibitory effect of atropine on nerve mediated responses relative to baseline conditions was significantly less in DBA (gray squares) at stimulations from 1 to 8 Hz compared to the effect in WT (black circles). (* significantly lower than WT at this frequency; # significantly higher than 1–4 Hz within DBA; ⇞ significantly higher than 1 and 2 Hz within DBA; ⇟ significantly different than 1, 2, 32 and 64 Hz within WT; $ significantly lower than 8–32 Hz within WT (N = 11 WT, N = 13 DBA, symbols indicate *p* < 0.05)).

After inhibition of the purinergic component of the neurogenic contractions, the amplitude of EFS responses was significantly decreased in both WT and DBA detrusors compared to baseline responses (Figures 9A,B). The effect of purinergic inhibition was frequency dependent in DBA detrusor tissue, but not in WT tissue. In DBA detrusors, the extent of inhibition was significantly less than that seen in WT detrusors at frequencies greater than 8 Hz (Figure 9C).

Inhibition of the cholinergic component decreased the amplitude of EFS responses in both WT and DBA bladder tissues compared to their respective baseline responses (Figures 9D,E). The effect of atropine generally increased with higher frequencies in both groups. At frequencies below 16 Hz in DBA tissue, the cholinergic component was significantly lower than that of WT tissue (Figure 9F).

**FIGURE 10:**
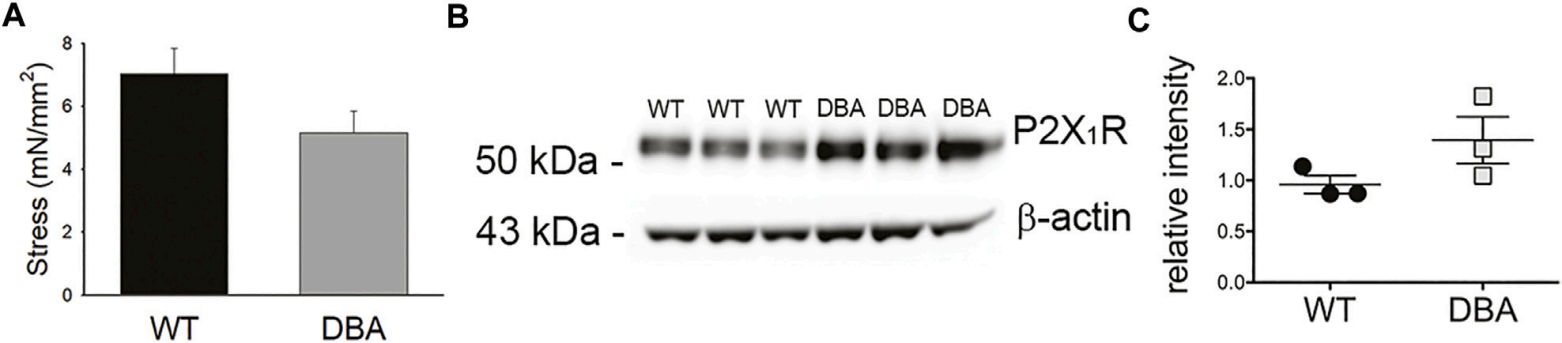
Purinergic responses of WT and DBA bladders. **(A)** Comparison of the contractile response of WT (black bar) and DBA (gray bar) bladder smooth muscle to the purinergic receptor agonist, αβmeATP (10 µM). Contractions of WT and DBA bladders were not significantly different under this condition (*N* = 11 WT, *N* = 13 DBA; *p* = 0.21). **(B)** Western blot comparing expression of the P2X_1_ purinergic receptor in WT and DBA extracts from bladder tissue devoid of mucosa. β-actin, shown in bottom panel, served as loading control. Molecular weights of mass standards (in kDa) are indicated at tick marks. **(C)** Quantitative data for WT (black circles) and DBA (gray squares) are graphed at right. The intensity of P2X_1_ receptor immunoreactivity, normalized by intensity of β-actin, was not different between groups (*N* = 3, *p* = 0.2).

To assess the capacity of WT and DBA detrusor smooth muscle to respond to purinergic stimulation, the amplitude of contractions induced by exogenous administration of the P2X_1_R agonist, αβ-meATP, was measured. No significant difference in the responses of WT and DBA bladders was observed (Figure 10A); furthermore, expression of the predominant purinergic receptor, P2X_1_R, was comparable in WT and DBA detrusor lysates (Figures 10B,C). These data indicate that smooth muscle contractions mediated by post-junctional purinergic activation are normal in DBA detrusor.

**FIGURE 11:**
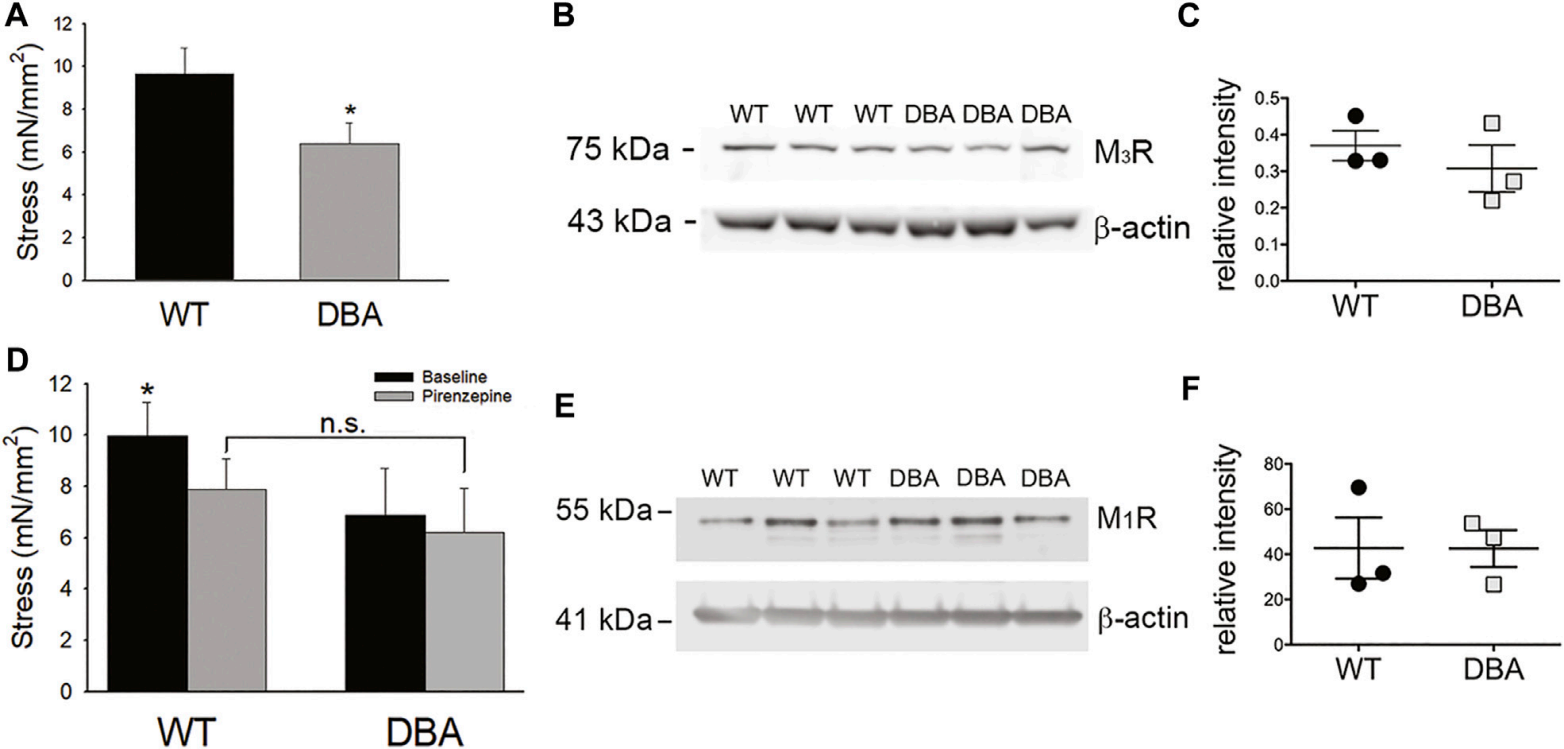
Cholinergic responses of WT and DBA bladders. **(A)** Contractile responses of DBA (gray bar) bladder smooth muscle to the muscarinic receptor agonist CCh, was significantly lower than WT (black bar) responses (*N* = 11 WT, *N* = 13 DBA, *p = 0.041). **(B)** Western blot comparing expression of M_3_R in WT and DBA bladder smooth muscle extracts, relative to β-actin loading control. The molecular weight in kDa of mass standards are shown by the tick marks. **(C)** Quantitative data indicate that muscarinic M_3_R expression in WT (black circles) and DBA (gray squares) detrusors was not significantly different (*N* = 3, *p* = 0.8). **(D)** Contractile responses induced by CCh in WT and DBA detrusors were generated before (black bars) and after (gray bars) inhibition of the M_1_R inhibitor with pirenzepine. In WT detrusors, CCh-evoked contractions were significantly inhibited by pirenzepine (*N* = 7; *p = 0.008), while contractions in DBA detrusors were not affected (*N* = 5, *p* = 0.31). In the presence of pirenzepine, the contractile response to CCh in WT was not different from DBA (*p* = 0.25). **(E)** Western blot comparing the expression of the facilitatory muscarinic receptor, M_1_R, in three WT and three DBA bladder muscularis extracts. Molecular weight in kDa of mass standards is indicated at tick marks. **(F)** The intensity of M_1_R immunoreactivity, normalized to the β-actin loading control, was not significantly different in WT (black circles) and DBA (gray squares) bladders (*N* = 3, *p* = 0.7).

While contractions induced in response to administration of the cholinergic agonist CCh were significantly lower in DBA detrusors than WT detrusors (Figure 11A), expression of the M_3_R was not different (Figures 11B,C). To resolve these divergent findings, responses to CCh were repeated in the presence of pirenzepine, a selective inhibitor of the muscarinic M_1_R. This receptor is expressed in bladder nerve fibers where it facilitates pre-junctional release of acetylcholine (Somogyi et al., 1994; Chess-Williams, 2002). In WT detrusors, the amplitude of the CCh response decreased significantly in the presence of pirenzepine to 70 ± 0.24% of the CCh response generated under baseline conditions (*p* = 0.033). In contrast, pirenzepine did not significantly alter the amplitude of CCh-induced contractile responses in DBA detrusors (90 ± 0.22% of the baseline response). Therefore, in the presence of pirenzepine, the CCh response in WT tissue was not different from DBA detrusors (Figure 11D). Moreover, the expression of the M_1_ receptor was comparable in WT and DBA detrusors (Figures 11E,F).

## Discussion

The motor protein Myo5a has been shown to facilitate directed vesicle motion along actin fibers; however, its role in bladder neurotransmission has not been previously explored. The data presented in this study establish that Myo5a is expressed by and localized in peripheral nerves of the urinary bladder. Notably, the well-characterized brain variant of Myo5a (which is abundant in neurons of the central nervous system) was not detected in detrusors of either DBA or WT animals, which are homozygous for, respectively, mutated and wild-type *Myo5a* alleles. The expressed Myo5a detrusor variants also differed by genotype. Moreover, compared to detrusors from WT mice, neurogenic contractions were attenuated in DBA detrusors. Together, these results indicated that Myo5a-dependent processes are crucial for efficient excitatory neurotransmission in the bladder, and demonstrated that molecular changes in Myo5a structure can be reflected in functional deficits in neurotransmission in this organ.

The seminal studies of Myo5a in the DBA mouse were largely confined to analysis of the mRNAs transcribed, and demonstrated that incorrectly spliced transcripts including Emv3 viral sequence could be identified in both brain and skin, but were more prevalent in skin. It was inferred that sufficient Myo5a brain variant protein was expressed in neurons to support normal development and neurological function in these mice, but that insufficient Myo5a skin variant proteins were expressed in melanocytes to support typical pigmentation (Seperack et al., 1995). This is consistent with our findings showing that the expression of the Myo5a brain variant incorporating exon B was not different between WT and DBA brains. Indeed, it has long been believed that dilute/gray fur color is the only phenotype of DBA mice resulting from their particular genetic alteration in the *Myo5a* gene. Compared with the more severe, and often lethal, phenotypes described in other *Myo5a* mutant strains, DBA mice do not display overt neurological dysfunction. However, we found reduced *Myo5a* expression in the detrusor of these mice, pointing to a previously unexplored autonomic peripheral neuropathy. Interestingly, elevated transcriptional initiation was also suggested in DBA compared to WT detrusor using the pan-Myo N-t realtime PCR assay, which targeted an exon boundary upstream of the alternative exon region. In contrast, the pan-Myo GTD assay, which targeted an exon boundary downstream of that region indicated reduced production of full-length Myo5a transcripts. This discrepancy implied that, despite a higher initiation of Myo5a transcription in DBA detrusor, fewer authentic processed transcripts were produced than in WT detrusor, presumably due to the misincorporation of viral sequence and premature termination of chimeric transcripts.

Our previous work challenges the viewpoint that Myo5a-dependent neurotransmitter transport is largely spared in the DBA mice. Our group previously provided evidence of subtle functional defects in peripheral innervation in DBA animals affecting the gastric fundus and the corpora cavernosa of the penis, organs in which nitric oxide and ATP are inhibitory neurotransmitters. We demonstrated impaired relaxation responses in DBA, attributing them to a reduced ability of Myo5a to effect transport of neuronal nitric oxide synthase (the enzyme that is activated and produces nitric oxide at the varicosity membrane), and of synaptic vesicles carrying ATP (Chaudhury et al., 2011, 2012; Chaudhury et al., 2014). Those data indicated a role for Myo5a in non-vesicular as well as vesicular transport of inhibitory neurotransmitters along peripheral nerve endings. The data presented here extend our observations to transport of excitatory neurotransmitters in efferent nerves of the urinary bladder, and additionally suggest that the exon D/F splice variants of Myo5a are involved in this process.

In rodent urinary bladder, the neurotransmitters acetylcholine and ATP are primarily responsible for parasympathetic contractions through activation of muscarinic M_3_ receptors and purinergic P2X_1_ receptors, respectively. Profound deficits in neurally-mediated responses were detected in DBA mice in this study, even though age, body or bladder weights, bladder tissue morphology, receptor-independent detrusor contractions, and the integrity of innervation were not different from those of WT mice. Given the comparable expression and function of purinergic receptors in DBA and WT bladder smooth muscle, the reduction in neurally-mediated contractile responses observed in DBA mice is consistent with a pre-junctional defect in release of ATP, rather than an altered smooth muscle response to direct purinergic receptor activation. Similarly, no differences between DBA and WT were detected in muscarinic receptor expression or in the contractile response to concurrent smooth muscle muscarinic receptor activation and presynaptic M_1_R inhibition. The lack of effect of pirenzepine in DBA detrusors likely reflects reduced acetylcholine release that is insufficient to provoke appreciable presynaptic facilitation. Together, these data provide evidence of reduced neurotransmission in DBA detrusors despite normal operation of smooth muscle contractile machinery.

Several aspects of neurotransmission were found to be defective in DBA detrusors. Both amplitude and AUC of nerve-mediated contractions were significantly lower in DBA compared to WT, and the temporal course of EFS-induced contractions was also remarkably different. With unaltered post-junctional contractility, the slower rate of the rising phase of contractions along with the longer time to peak and decay time constant of DBA contractions likely reflect a reduced amount and dampened kinetics of neurotransmitter release. Furthermore, the extent to which the purinergic and cholinergic components of neurogenic contractions were reduced in DBA detrusors displayed a complementary pattern that depended on the stimulation frequency. In particular, the effect of purinergic inhibition was similar in DBA and WT detrusors at lower frequencies, but became significantly reduced at higher frequencies (16–64 Hz). In contrast, cholinergic inhibition was less efficient in DBA than WT at frequencies up to 8 Hz, but similar to WT at higher frequencies of stimulation. These data are fully consistent with the diminished amplitude of EFS-induced contractions over the entire range of frequencies observed in DBA in the absence of receptor antagonists and suggest a frequency-dependent susceptibility of neurotransmitters to altered Myo5a splice variants in these mice.

The specific contribution of individual Myo5a splice variants to bladder neurotransmission have not been addressed in this initial study. However, one important implication of the data we present here is that variants with exons D/F but which lack exon B, are required for the Myo5a-dependent transport of ATP- and acetylcholine-containing synaptic vesicles in bladder motor nerve varicosities. To our knowledge, this is the first indication of a role for these Myo5a variants in excitatory neurotransmission.

Identification of the splice variants comprising the population of Myo5a proteins in the detrusor may be relevant for predicting their interacting Rabs (the membrane-bound GTPases which regulate all aspects of vesicle trafficking) and ultimately their neurotransmitter-containing cargo. For example, exon D has been shown to mediate interactions between Myo5a and Rabs 8a and 10 (Roland et al., 2009; Lindsay et al., 2013), which in adipocytes facilitate Myo5a-dependent translocation of Glut4-containing vesicles to the plasma membrane (Chen et al., 2012; Sun et al., 2014). Moreover, exon F is required for the Myo5a-dependent transport of melanosomes, forming a tripartite complex with melanophilin and the melanosome membrane binding protein, Rab27a (Lambert et al., 1998; Au and Huang, 2002; Fukuda et al., 2002; Wu et al., 2002). Additional roles for exon F are emerging from studies conducted in other cell types, perhaps reflecting the structural influence that exon F exerts on Myo5a conformation (Figure 2; Seperack et al., 1995; Au and Huang, 2002) which could markedly affect GTD-cargo interactions. Effector proteins such as MyRIP (myosin and Rab27a interacting protein) and rabphilin bind to Rab27a and Myo5a in endocrine cells (Desnos et al., 2003; Brozzi et al., 2012). In endothelial cells, multimers of the hemostatic protein, von Willebrand factor, are secreted from specialized granules (Weible-Palade bodies) in a process involving Rab27a, MyRIP, and the Myo5a variants ABCEF and ABCDEF (Rojo Pulido et al., 2011). In the urothelium, Myo5a complexes with Rab27b to tether uroplakin-positive fusiform vesicles to cortical actin filaments in umbrella cells (Wankel et al., 2016). Also, several Rabs (3a, 10, 11, 14, and 27b) which bind to Myo5a either directly or through an accessory protein, associate with synaptic vesicles in neurons (Pavlos et al., 2010; Lindsay et al., 2013). Our future work will examine the interactions of bladder Myo5a variants with these vesicle constituents to discern their mechanisms of action in neurotransmitter secretion.

## Conclusion

Myo5a is involved in multiple and sequential effector interactions during the transport, tethering and release of neurotransmitter-containing vesicles at nerve varicosities. In murine bladder nerves, the shorter Myo5a variant characteristic of brain neurons (ABCE), was not detected while longer variants typical of skin (ACDE, ACEF, ACDEF) predominated. Reduced total Myo5a expression, as well as limited production of these longer splice variants, are factors implicated in deficiencies of neurogenic smooth muscle contraction in DBA bladder described here. This first study of Myo5a splice variants in peripheral motor nerves in the bladder may have broader implications for Myo5a-dependent neurotransmission in other visceral organs, with the array of splice variants expressed, as well as the ratios among them, reflective of the particular functional requirements of each organ and cell type.

## Data Availability

The original contributions presented in the study are included in the article/Supplementary Material. Further inquiries can be directed to the corresponding author.

## Supplementary Material

Supplementary Figure S1 Negative control for immunofluorescence microscopy. Confocal microscopy image of mouse bladder tissue sections (12 µm) in which the primary antibody was omitted (left image) showed no green fluorescent signal compared to tissue incubated for the same amount of time with mouse anti-Synaptophysin antibody (Abcam SY38, 1:10; right image). Alexa Fluor 488 donkey anti-mouse IgG (H + L) highly cross-adsorbed secondary antibody (A-21202, RRID AB_141607; Thermo-Fisher) was applied on both preparations at dilution of 1:2000. The superimposed differential interference contrast image (DIC) delineates tissue morphology (Magnification ×40, scale bar 50 µm).

Supplementary Figure S2 Myo5a localization and variant expression in bladder mucosa. **(A)** Image from confocal microscopy of bladder sections stained with Myo5a antibody (red staining) and counterstained with urothelial marker uroplakin-3 (UPIII, green staining). Differential interference contrast (DIC) image was superimposed to visualize tissue anatomy. Myo5a showed abundant immunoreactivity in nerves fibers running within the muscle bundles as well as in the *lamina propria*. Less intense staining for Myo5a was detected in the urothelial layers as shown by the merged image (Scale bar = 200 µm; Mag ×20). **(B)** cDNAs from bladder mucosa total RNA of WT (black circles, *N* = 4) and DBA animals (gray squares, *N* = 3) were assayed by RT-PCR in triplicate for Myo5a exon B or exon F, as indicated, with their specific TaqMan assays, and for 18S rRNA as an internal control. Expression was determined by the ∆∆Ct method and the average % intensity, shown by the horizontal line, ± SEM was graphed relative to WT. Expression of exon B was not detected (nd) in either WT or DBA mucosa; exon F was detected but expression did not differ by genotype (*p* = 0.52). **(C)** cDNAs from mucosae of WT (*N* = 3) and DBA (*N* = 3) animals were used for nested PCR with primer pair 3 which spans alternative exon D sequence. Products were digested with Ban II to indicate the presence of exon D, and electrophoresed on a 2% MetaPhor agarose gel with a 50 bp mass standard ladder to assess fragment sizes. The mobility of bands without (-D) and with (+D) exon D are shown at the right. **(D)** The intensity of each band as a percentage of the total intensity in each lane was determined, and the average % intensity ± SEM of +D products, shown by the horizontal lines is graphed for all replicates of each genotype. There was more exon D-containing PCR product seen in WT mucosa (black circles) compared to DBA mucosa (gray squares) (**p* < 0.05). **(E)** Nested PCR was also performed with primer pair 4, in which the reverse primer is within exon F. Products were digested with Ban II and electrophoresed with a 100 bp mass standard ladder, to indicate the proportion of exon F-containing transcripts that also contain exon D. The second and third lanes of this gel contain an undigested aliquot of WT and DBA mucosa from these PCR reactions, respectively. The mobility of bands without (−D +F) and with (+D+F) exon D are indicated at the right. The intensity of each band as a percentage of the total intensity in each lane was determined, and the average +D+F band intensity ± SE is graphed for all replicates of each genotype in panel **(F)**. Black circles represent WT mucosa, gray squares, DBA mucosa. There was more exon D-containing PCR product seen in WT mucosa compared to DBA mucosa (**p* < 0.05).
