# Supplementary figures and images for: Myosin 5a in the Urinary Bladder: Localization, Splice Variant Expression, and Functional Role in Neurotransmission
Source: Front Physiol. 2022 Jul 1;13:890102. doi: 10.3389/fphys.2022.890102 (PMC9284544; doi:10.3389/fphys.2022.890102)

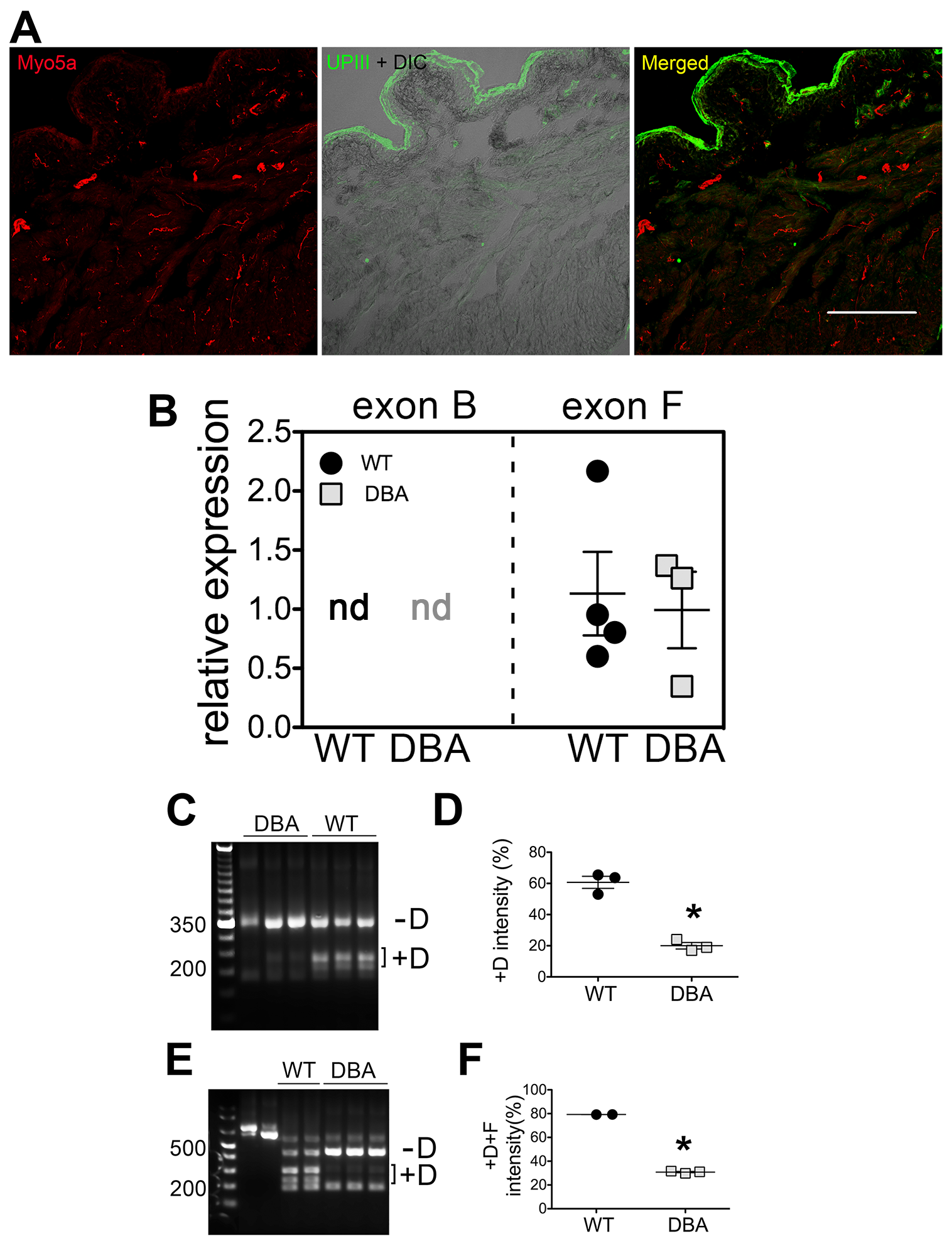

Supplement: Supplementary file 1 [file Image2.tif]

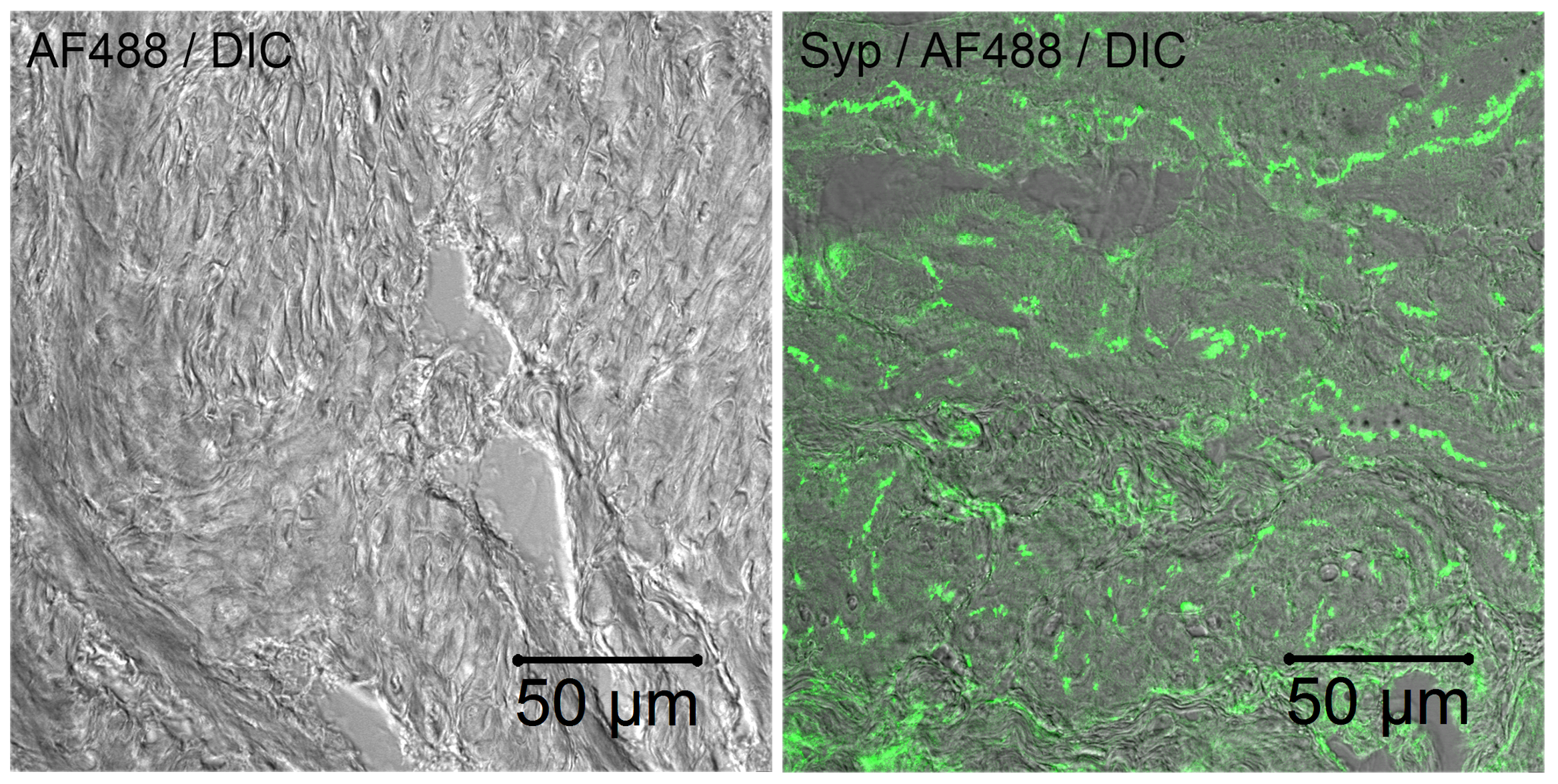

Supplement: Supplementary file 2 [file Image1.tif]
